# Comparison of Cortisol Levels in the Hair of Male European Roe Deer at the Beginning and End of the Stalking Hunting Season

**DOI:** 10.3390/ani14223184

**Published:** 2024-11-06

**Authors:** Katarzyna Dziki-Michalska, Katarzyna Tajchman, Patrycja Staniszewska, Aneta Strachecka

**Affiliations:** 1 Department of Animal Ethology and Wildlife Management, Faculty of Animal Sciences and Bioeconomy, University of Life Sciences in Lublin, Akademicka 13, 20-950 Lublin, Poland; katarzyna.michalska@up.lublin.pl; 2 Department of Invertebrate Ecophysiology and Experimental Biology, Faculty of Animal Sciences and Bioeconomy, University of Life Sciences in Lublin, 50A Doświadczalna Street, 20-280 Lublin, Poland; patrycja.staniszewska@up.lublin.pl (P.S.); aneta.strachecka@up.lublin.pl (A.S.)

**Keywords:** *Capreolus capreolus*, environmental pressure, hair cortisol

## Abstract

Roe deer (*Capreolus capreolus*), as a representative of the Cervidae family, are particularly sensitive to negative environmental stimuli due to their need to maintain increased vigilance during feeding. Intensive hunting seasons are undoubtedly also a stressful factor for this species. The aim of this study was to analyze the cortisol levels in the hair of male roe deer collected at the beginning and the end of the hunting season in eastern Poland. In total, hair samples from 26 individuals collected in May and 31 individuals collected in September were analyzed. The concentration of cortisol in the hair was determined using the EIA kit. It was shown that the concentration of cortisol was significantly higher in samples collected in September compared to those collected in May (Pr. > |t| = 0.0017). Moreover, the age of animals and carcass mass did not significantly affect the concentration of the tested hormone. In summary, the concentration of cortisol in the fur of male roe deer depended on the season and was not influenced by the carcass mass or age of the animals studied.

## Simple Summary

Hunting is one of the environmental factors that can affect the welfare of game animals. Glucocorticoid hormone measurement can be a reliable indicator of stress response. To assess the intensity of the endocrine response, the level of cortisol concentration in the hair of male European roe deer obtained at the beginning and the end of the hunting season in eastern Poland was analyzed. It was shown that the cortisol concentration was significantly higher in males obtained at the end of the hunting season, but not affected in the carcass mass of the animals studied. To sum up, the hunting season or other factors (climatic or intra-population) influence the stress levels of young male European roe deer.

## 1. Introduction

The European roe deer (*Capreolus capreolus*) is the most numerous species of cervid in Poland. The Central Statistical Office estimates its population at 918,500 individuals [1]. Compared to 1990, the population of this species has almost doubled, and consequently, it is increasingly hunted in Poland [1]. Researchers indicate that animals from the cervid group are characterized by high sensitivity to negative environmental factors [2,3,4,5]. There is no doubt that the high reactivity of roe deer, in combination with the multitude of exogenous negative stimuli, has a negative impact on their health and individual conditions [6]. The animals are not indifferent to stress, which can lead to several undesirable effects [7,8]. Each negative factor, both endogenous and exogenous, causes the release of glucocorticosteroids from the adrenal cortex into the blood [9]. They enable the animal organism to take rapid actions aimed at eliminating and avoiding the impact of the stimulus. The body’s quick and short-term response to stress has a mobilizing effect on the body and is important for survival in times of danger [10]. The negative effects of the hypothalamic–pituitary–adrenal (HPA) axis include, among others, decreased individual immunity [11,12] and, consequently, increased susceptibility of the organism to diseases [13]. High cortisol (CORT) levels are associated with behavioral, physiological, and nutritional disorders [14,15]; therefore, the analysis of the impact of predictable negative stimuli on wild animals is justified [16]. Hunting, as an element of reasonably conducted game management, has an impact on the welfare of game animals [4,5].

A sufficiently strong and short-lasting negative stimulus activates the HPA axis [17,18], which translates into a release of glucocorticoids and mobilizes the body to the appropriate behavioral and physiological response, which is aimed at restoring the body to a state of complete balance [19,20,21]. The animal’s body’s response to stress leads to increased attention, cardiac minute capacity, resorption, and catabolism [22]. A commonly used indicator of stress response intensity is cortisol (CORT) [4,5,23]. CORT is considered a reliable indicator of stress, but its concentration is species- and individual-dependent [23,24,25]. CORT and its metabolites are most often measured in the blood, plasma, saliva, feces, and urine [26]. Nevertheless, the results of the tests only concern the body’s reactions occurring in a relatively short period, from a few minutes to a maximum of two days before the sample was taken [27,28,29]. In the assessment of repeated or chronic stress, it has been shown that the CORT level in the hair and fur of animals is a reliable indicator [30,31,32,33,34]. The measurement of cumulative CORT in hair is a valuable indicator of chronic stress in wild animals [35,36,37] due to its incorporation into the hair shaft in the active growth phase [37], which lasts for a longer period [37,38]. Unfortunately, we do not have a full picture of this process [32,39]. Since the discussed cycle concerns free-living animals, there are no accurate and precise data on CORT accumulation or hair growth rate in the context of species differences, and this may result in certain limitations in the interpretation of the data obtained by researchers [40].

Undoubtedly, hunting, as a highly stressful stimulus, may be one of the factors that affects the well-being of cervids [9]. Studies conducted on red deer have shown that hunting results in, in addition to an increase in cortisol levels, changes to the concentration of β-endorphin and the depletion of carbohydrate resources, which is classified as indicating extreme stress [41]. However, stalking hunting is characterized by a stress response of lower intensity than, for example, hunting with hunting dogs or with the participation of many hunters [28]. Therefore, the cyclicality of hunting seasons may cause recurrent chronic stress in cervids [9]. Nevertheless, it is worth noting that, in wild Cervus canadensis, no increase in glucocorticoids in feces was confirmed in connection with the number of hunting days [29]. This may be related to adaptation to repeated negative factors and, consequently, to the plasticity of cervid behavior, which is aimed at avoiding strongly negative stimuli [25]. Studies conducted on roe deer obtained via stalking have shown a large range of results, which may indicate that individual variability (resistance to stress, time of exposure to the stimulus) may be decisive in the intensity of the stress reaction [4]. It should be noted, however, that increased physical activity (caused by, for example, tracking and stalking during individual hunting) causes a slight increase in cortisol concentration in a situation where trauma (e.g., collision with a car) results in a disproportionately higher concentration of this glucocorticoid [42]. In Poland, male roe deer are obtained only during the hunting season, which begins on 11 May and lasts several months—until 30 September of the same year. During this period, bucks are obtained only and exclusively through stalking hunts. Stalking hunts are usually performed in the early morning or evening hours, but not during times of darkness, which means from one hour before sunrise to one hour after sunset. These are periods of the day when animals are very active in eating and moving around. Intensive stalking hunts taking place during this time can have a negative impact on the roe deer population [4,5]. Therefore, this study aimed to assess the effects of stalking hunts on the level of long-term stress in male European roe deer (*Capreolus capreolus*) by comparing the levels of cortisol in the hair of animals obtained at the beginning and at the end of the stalking hunting season, depending on age and carcass mass.

## 2. Materials and Methods

### 2.1. Experimental Design

Roe deer were obtained in accordance with Polish Hunting Law and the principles of individual selection during the hunting season [43] for individual hunts in the Lubartów Forest District of the Game Breeding Centre (GBC) in Kozłówka and Rawityn in May and September 2023. Hunting exploitation of European roe deer in Poland is permitted at a density of 1.5 individuals per 100 ha of hunting district area. The number of young individuals is calculated as 70% of the number of females. The planned harvest of roe deer depends on the density, number of young individuals, habitat possibilities, and long-term hunting management plans. The harvest structure is assumed to be as follows: females—up to 50%, males—up to 50%, and fawns—up to 30%. The criteria for the selection of male roe deer are as follows: individuals with first antlers up to 70% of the total number of males planned for shooting (males with first antlers in the shapes of buttons, straight pointed antlers without side branches, antlers with side branches up to 2 cm long); males with 2nd–4th antlers (all antlers with a smaller number of side branches than regular antlers, three side branches on each beam over 3 cm long), min. 30% of the total number of males scheduled for shooting; and males with 5th antlers and older, regardless of the shape of the antlers [43]. The shooting of animals had a selective purpose and was carried out by qualified hunters—employees of the Lubartów Forest District. In order to obtain animals, they conducted single-person hunts on foot without the participation of hunting dogs to minimize the strength of negative stimuli associated with the procedure. The shooting took place in the early morning hours. The animals died immediately after the first precise shot.

The Lubartów Forest District is located in Central and Eastern Poland (51°27′ N, 22°29′ E). Poland has a temperate, warm, transitional climate. The forests of the Forest District are located mostly in the IV Mazovian-Podlaskie Region, the 5th district of the Podlaskie Lowland, and the Siedlce Upland. The terrain is not very diverse and is characterized by flatness. The annual rainfall is 552 mm, and the average annual temperature is +7.7 °C. The forest district is characterized by a large diversity of habitats and soil fertility. The forest cover of the region is estimated at 24.9%, with 49% of the area covered by coniferous species and 38% covered by mixed forests with deciduous species [44]. In the area of the forest district, the density of red deer (*Cervus elaphus*) is 16.03/1000 ha of forest area, that of roe deer (*Capreolus capreolus*) is 2.08/1000 ha of the total area, and that of moose (*Alces alces*) is 10.15 individuals per 1000 ha of forest and marsh area [44].

GBC Kozłówka has a total area of 10,757 ha, of which 5945 ha (55%) is forests, and GBC Rawityn has an area of 8080 ha, of which 3666 ha (45%) is forests [44]. In the 2022/2023 stalking hunting season, 32 male roe deer were shot in is each area (64 individuals in total in both GBCs) from May to September by 11 hunters (data from the Lubartów Forest District).

Ethical review and approval did not apply to this study because biological material was collected after the hunting procedures. Samples from roe deer were harvested during the hunting period (from 11 May to 30 September 2023) in accordance with the Polish Hunting Law, Annex to Resolution No. 57/2005 of 22 February 2005.

### 2.2. Sampling

The individuals harvested in the study were obtained between 11 and 25 May (in the first two weeks after the start of the male hunting season) and between 15 and 30 September (in the last two weeks of the hunting season). On both dates, only summer hair of reddish-brown color was collected from the carcasses of animals. It was cut with laboratory scissors from the skin in the dorsal–caudal region. Full-length hair samples were frozen within the shortest possible time after collection (up to 15 min) and kept at −25 °C until the tests.

### 2.3. Laboratory Analysis

The extraction methodology was modified from that of Burnett et al. [45]. The collected hair was washed with warm water and dried for one day at room temperature (24 °C) in ambient air, and then washed in 5 mL isopropanol for 3 min and dried for 5 days (24 °C in ambient air). The full-length hair was cut to 1–2 mm in length using laboratory scissors, and then 70 mg of trimmed hair was put into a glass vial. Three-and-a-half methanol (Sigma-Aldrich, Poznań, Poland) was added, and vials were incubated for 18 h at 37 °C with shaking. After incubation, the supernatant was filtrated using 30 mm diameter syringe filters with a porosity of 0.22 mm (Alfachem, Poznań, Poland) to separate the liquid phase, then placed into disposable glass culture tubes. The supernatant was evaporated at 45 °C in ambient air until completely dry. The extracted cortisol samples were assayed using a commercially available EIA kit (Cortisol Enzyme Immunoassay Kit, K003-H5, Arbor Assays, Ann Arbor, MI, USA) according to the manufacturer’s instructions with the reagents provided. As recommended by the test manufacturer, sensitivity was calculated by comparing the optical density (OD) for the blank and the highest concentration of the standard. The detection limit was determined at two standard deviations from the blank along the standard curve. Sensitivity was set at 27.6 pg/mL. The limit of detection for the assay was determined similarly, comparing the OD values for twenty runs each of the blank standard and the low-concentration sample. The detection limit was determined to be 45.4 pg/mL. The age of the animals was determined post mortem using the Eidmann method, which involves assessment based on the layers of dentine deposited in the canal of the first pair of incisors I1, the characteristic features of the dentition, the stage of development, and the replacement of primary teeth with permanent ones [44].

### 2.4. Statistical Analyses

The statistical analysis used GLM and CORR procedures of SAS software (Statistical Analysis System, 9.4, 2013). The normality of data distribution was assessed using the Shapiro–Wilk test. The significance of differences between CORT content means was verified using multivariate analysis of variance with Tukey’s test (proc GLM). In the model, the month of sampling (2 levels—September and May), animal age (2 levels: 3–4 years old and 5–6 years old), and interaction of sampling month and age were used as qualitative factors, while the carcass mass of the animals was used as a quantitative factor. Additionally, the relationship between animal age and CORT concentration was examined using Pearson correlation. Results were presented using basic statistics and the significance of differences between means at *p* ≤ 0.05.

## 3. Results

The CORT concentration was significantly higher in September compared to May (Pr. > |t| = 0.0017) (Table 1). The age of the animals and the interaction of the month of sampling and age did not significantly affect the CORT concentration in the hair of the studied animals.

The animal carcass mass did not affect CORT concentration (F = 0.41, Pr. > F = 0.53; t = 0.64, Pr. > |t| = 0.53) using the GLM linear model. The Pearson correlation between roe deer carcass mass and CORT concentration was −0.019 and was not significant (Pr. > r = 0.88) (Table 1).

## 4. Discussion

The average CORT concentration in hair collected in May in both age groups of male roe deer was almost identical, and it was similar in September. The results of our analysis of hair collected in May are consistent with the average results obtained by Ventrella et al. [3] in June. However, the CORT concentration in hair collected in September was different [3]. Our study has indicated a much higher CORT concentration in roe deer hair at the end of the hunting season and after the mating season, which falls at the turn of July and August. The same results have already been reported in other species [46,47,48]. In turn, studies conducted in Italy on roe deer that died as a result of collisions with cars did not show any correlation between the month of sampling and the CORT concentration in the fur of these animals, and the levels were slightly higher than those obtained in our own research [37].

It should be emphasized that, at present, there are no reference values to which the results obtained by the authors of this study could be related. However, other researchers have shown much higher CORT concentrations in the hair of red deer [49] or elk [39]. The differences are most likely due to species differences, as well as environmental factors such as climate, increased predation pressure, or population density in the area where the population lives [49]. Franchini et al. [37] indicated that higher CORT concentrations in late summer and autumn may be characteristic of areas with a cold climate, and Huber et al. [50] confirmed in their studies higher CORT concentrations in months characterized by lower temperatures. Rakic et al. [51] emphasized that the CORT concentration in the fur fluctuates throughout the year and is dependent on the timing of hair coat changes; it increases when the hair is not growing (resting phase), which has been confirmed by studies conducted on grey wolves [52] and American hares (*Lepus americanus*) [53].

Our own studies, however, indicate that the CORT concentration was significantly higher in September compared to May. The basic factor that may influence the increase in stress levels in male roe deer, apart from the ongoing hunting season, is the mating season. Territorial behavior in European roe deer emerges with the arrival of spring, reaching its peak during the rut. At that time, males display intensified territorial behavior, characterized by increased aggression [54], which may have an impact on our results. Studies conducted so far on cervids confirm that rut is a particularly stressful period for cervids [28,50,55]. The increase in cortisol concentration in the fur may also be influenced by negative environmental factors, including climate, ambient temperature, or the high density of the species in a given area [12,49,56,57,58]. In the study region, the average temperatures for the months covered by the study were: May: 9–16 °C, June: 16–26 °C, July: 15–23 °C, August: 10–32 °C, and September: 9–19 °C [59]. It can therefore be concluded that the temperature value for the above-mentioned months could have been an additional negative factor causing heat stress [60], because in the case of moose (*Alces alces*), the upper- temperature limit that does not cause the above stress is 14 °C in the summer months [61], moose being an excellent example of a species sensitive to maximum temperatures in each season [60].

An important factor influencing the welfare of roe deer is the density of the red deer (*Cervus elaphus*) population in its habitat. Many authors indicate that a high density of this species may negatively affect the population of roe deer, primarily due to food competition [62,63,64]. It should therefore be considered that potential interactions of these two species may lead to increased CORT secretion in roe deer, especially when both species share the same habitats [37]. However, in the study area, the density of red deer probably does not have a negative impact on the population of European roe deer, as it was at the level of 16.03 individuals per 1000 ha of forest area [44]. According to the recommendations of the Polish Hunting Association, it should oscillate between 15 and 35 individuals per 1000 ha of forest area—such values allow for rational hunting management [43].

It is worth emphasizing that lower CORT concentrations in various species, including roe deer, are typical for warmer months—CORT concentration increases from early spring to late summer [3,64,65], which contrasts our results. It is also important to note that a high concentration of androgens during the rut in roe deer may affect the CORT concentration caused by stress [66,67], although the relationship between CORT and testosterone may be apparent due to seasonal fluctuations in both of these hormones [3].

The results obtained herein confirm the lack of a relationship between the carcass mass of animals, age, and CORT concentration in the hair, which has already been noted [40,49]. This allows us to conclude that the factors that have the strongest impact on the welfare of roe deer are primarily environmental factors, including animal disturbance during hunting seasons. Considering the cyclical repetition of this negative stimulus, it may cause chronic stress in free-living animals [9]. Gentsch et al. [28] indicated that individual hunting is less stressful than, for example, the death of an animal due to a road accident, but little is known about long-term stress in wild animals. The mere presence of hunters in the hunting ground is a negative stimulus for cervids and affects their welfare [5,9]. Studies conducted by Bateson and Bradshaw [41] indicate that the increasing concentration of cortisol in the feces of red deer is positively correlated with the intensity of hunting pressure. A lack of such a correlation was demonstrated by Ensminger et al. [28] in studies conducted on wild *Cervus canadensis*. Therefore, determining the concentration of CORT in the fur of game animals offers a reliable indicator for tracking its trends and monitoring the welfare of these animals [39,51].

## 5. Conclusions

In summary, the cortisol concentration in the hair of male European roe deer was significantly higher in animals obtained at the end of the stalking hunting season. Disturbing animals during the hunting season may be one of the factors that generates chronic stress in them, especially if the negative stimulus is repeated over a longer period of time. However, it may also be an effect of the season, but it did not correlate with the carcass mass or the age of the animals. The results obtained and analyzed herein can be a valuable source of information in the context of deer welfare. Still, this is preliminary research and should be expanded upon in subsequent years, along with other methods of assessing welfare.

## Figures and Tables

**Table 1:** Descriptive statistics of the analyzed parameters for animals harvested at the beginning and at the end of the hunting season.

Month of Sampling	N	Age (Years)	Mean Carcass Mass (kg)	Mean Cortisol Concentration (pg/mg)	Standard Deviation	Standard Error	Coefficient of Variation
May	15	3–4	16.866	0.395 ^a^	0.016	0.004	3.929
	11	5–6	18.000	0.397 ^ab^	0.006	0.002	1.407
September	17	3–4	15.882	0.418 ^ab^	0.034	0.008	8.045
	14	5–6	18.571	0.428 ^b^	0.041	0.011	9.658

^a, b^—means in the column differ significantly at *p* ≤ 0.05 (Tukey test).

## Data Availability

The data that support the findings of this study are available upon request from the corresponding author.

## Abbreviations

CORT: cortisol; A: age; CM: carcass mass.
